# Attention problems and cortical maturation in a large longitudinal sample of youths: The importance of accounting for sex differences

**DOI:** 10.1073/pnas.2605729123

**Published:** 2026-05-18

**Authors:** Shannon D. O’Connor, Robert Loughnan, Jonathan Ahern, Chun Chieh Fan, Robert R. Althoff, Hugh Garavan, Alexandra Potter, Matthew D. Albaugh

**Affiliations:** ^a^Department of Psychiatry, University of Vermont, Burlington, VT 05401; ^b^Center for Population Neuroscience and Genetics, Laureate Institute for Brain Research, Tulsa, OK 74136; ^c^Department of Cognitive Science, University of California San Diego, La Jolla, CA 92023; ^d^Department of Radiology, University of California San Diego, La Jolla, CA 92023

**Keywords:** neurodevelopment, ADHD, attention problems, adolescent, neuroimaging

## Abstract

Delayed age-related cortical thinning has been proposed as a biomarker of attention-related psychopathology and attention-deficit/hyperactivity disorder (ADHD), but these findings have not been adequately replicated in large, longitudinal samples with sufficient power to account for potential confounds such as co-occurring psychopathology and sex differences in neurodevelopment. Here, we examined whether previously reported associations between attention problems and delayed cortical thickness development could be replicated in a large, longitudinal cohort of youths. Leveraging data from the Adolescent Brain Cognitive Development Study, linear mixed-effects models were used to assess the association between parent-reported attention problems (AP) on the Child Behavior Checklist and age-related cortical thickness change (N = 26,496 MRI scans from 11,025 unique participants). Secondary analyses examined the association between a polygenic risk score for ADHD and cortical thickness development. In initial sex-pooled analyses, we observed associations between AP and reduced rates of cortical thinning (β = 00594 to 0.0145, FDR-adjusted *P* < 0.05). However, when an age × sex interaction term was included in the model, these associations were no longer evident (all FDR-adjusted *P* > 0.05 across ROIs). Follow-up sex-stratified analyses revealed no significant age × AP interactions on cortical thickness in males or females. Further, there was no evidence of genetic liability for ADHD being associated with reduced age-related cortical thinning. Taken together, these findings suggest that previously reported associations between AP and delayed cortical thinning likely reflect unaccounted-for sex differences in neurodevelopment rather than AP-specific maturational delays, questioning the utility of cortical maturation patterns as diagnostic biomarkers for ADHD-related behavior.

Delayed cortical thickness maturation during childhood and adolescence, as measured using magnetic resonance imaging (MRI) techniques, has been linked to attention-deficit/hyperactivity disorder (ADHD) and dimensionally assessed attention problems. In an influential PNAS publication, Shaw et al. (1) were the first to report delayed cortical maturation among youths diagnosed with ADHD relative to typically developing controls. In their landmark study of 223 children with ADHD, apparent maturational delays were most prominent in prefrontal cortices where youth with ADHD, on average, lagged by several years compared to controls. Similarly, dimensional assessments of attention problems have been associated with delayed rates of age-related cortical thinning across both clinical and typically developing samples, with prefrontal regions again showing the strongest effects (2, 3). Such findings suggest that dimensional variation in attention problems reflects, at least in part, the same underlying liability captured at the clinical extreme, consistent with genetic evidence for continuity across normative and clinical symptom levels (4).

While these reports of delayed cortical maturation were compelling—seemingly paralleling ADHD-related behaviors often described as developmentally delayed—subsequent replication efforts have been sparse. Nonetheless, the narrative linking attention problems to delayed cortical maturation remains widespread in research and clinical contexts. Since then, significant advances have been made with respect to image processing, cortical surface reconstruction, quality control procedures, and appropriate modeling of age-related cortical thickness change. For example, methodological refinements have revealed that the inverted-U developmental thickness trajectory reported by Shaw et al. (1) and others was likely artifactual, with age-related cortical thinning being largely monotonic from early development onward (5). Moreover, recent work has demonstrated that reproducible brain–behavior associations require much larger samples than previously thought, often necessitating thousands of participants (6). The advent of large, publicly available longitudinal neuroimaging studies provides the opportunity to test whether prior reports of ADHD symptomatology and delayed cortical thickness maturation are replicable.

The present study addresses these methodological concerns, as well as the lack of large-scale replication efforts, using data from the Adolescent Brain Cognitive Development Study. Leveraging this large dataset, we first test whether well-validated, dimensional assessments of attention-related psychopathology are associated with cortical thickness maturation. We then investigate whether observed associations are robust to co-occurring psychopathology and to established sex-related differences in cortical thickness development, with males generally evidencing slower rates of age-related cortical thinning (7). Last, given that polygenic risk for ADHD predicts dimensional measures of attention problems in population-based samples of youth—indicating shared genetic underpinnings across clinical and normative symptom levels (4)—we test whether genetic liability for ADHD moderates age-related cortical thinning.

## Results

**Fig. 1.**
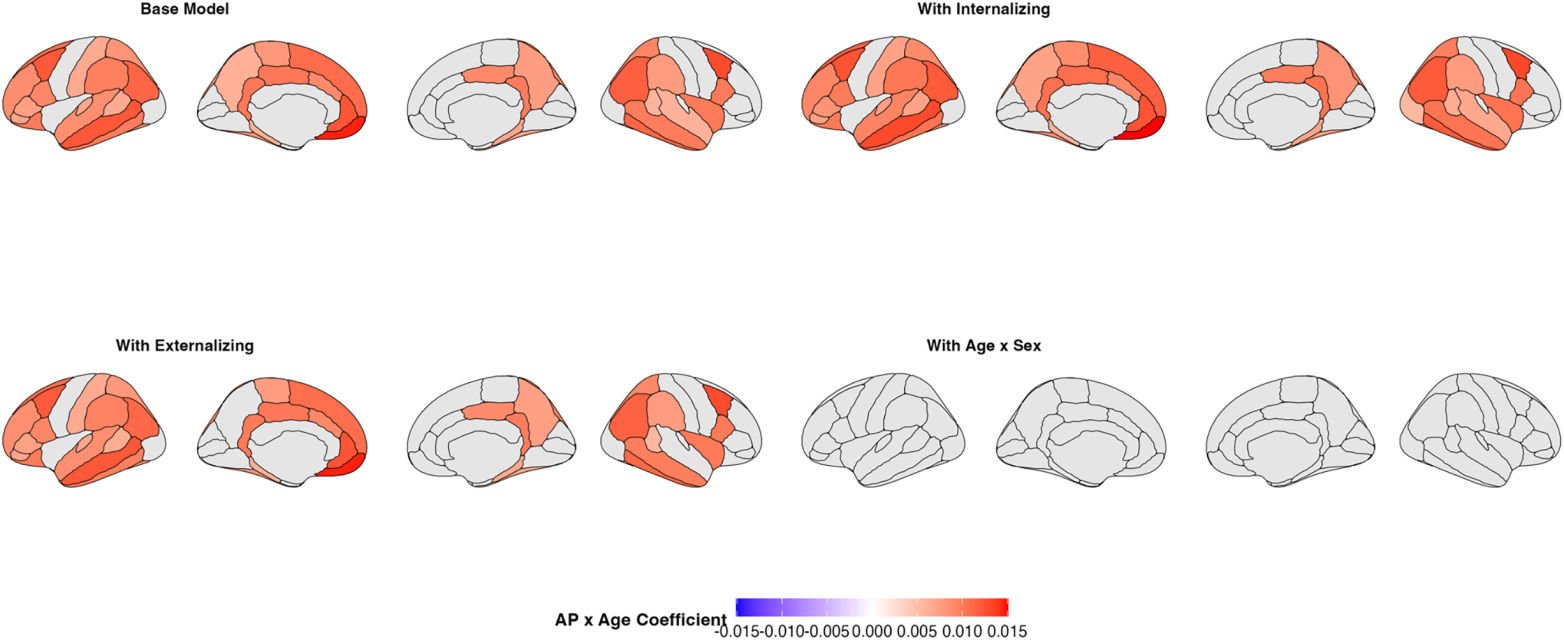
Shown above are standardized coefficient maps for significant age × AP effects in the initial model (*Top* *Left*), the model that included internalizing (*Top* *Right*), the model that included externalizing (*Bottom* *Left*), and the model that included age × sex (*Bottom* *Right*). In the initial sex-pooled model, the following 40 ROIs showed a significant, positive age × AP effect: banks of the superior temporal sulci (*Left*: 0.00664, *P* = 0.0232; *Right*: 0.00594, *P* = 0.0357), caudal anterior cingulate (*Left*: 0.00903, *P* = 0.00712), caudal middle frontal (*Left*: 0.0122, *P* = 0.00203; *Right*: 0.0129, *P* = 0.00147), fusiform (*Left*: 0.00837, *P* = 0.0187; *Right*: 0.00699, *P* = 0.0396), inferior parietal (*Left*: 0.0114, *P* = 0.00124; *Right*: 0.0118, *P* = 0.00124), inferior temporal (*Left*: 0.00993, *P* = 0.00414; *Right*: 0.0114, *P* = 0.00124), insula (*Right*: 0.0101, *P* = 0.0396), isthmus cingulate (*Left*: 0.0102, *P* = 0.00124; *Right*: 0.0099, *P* = 0.00147), lateral orbitofrontal (*Left*: 0.01, *P* = 0.0195), medial orbitofrontal (*Left*: 0.0145, *P* = 0.00218), middle temporal (*Left*: 0.0123, *P* = 0.00114; *Right*: 0.00994, *P* = 0.00218), paracentral (*Left*: 0.00798, *P* = 0.0232), parahippocampal (*Left*: 0.00618, *P* = 0.0232; *Right*: 0.00692, *P* = 0.0232), pars opercularis (*Left*: 0.00929, *P* = 0.00887; *Right*: 0.0101, *P* = 0.00351), pars orbitalis (*Left*: 0.00699, *P* = 0.0439), pars triangularis (*Left*: 0.00742, *P* = 0.0396), postcentral (*Left*: 0.00673, *P* = 0.0396), posterior cingulate (*Left*: 0.0103, *P* = 0.00124; *Right*: 0.00902, *P* = 0.00643), precuneus (*Left*: 0.00619, *P* = 0.0419; *Right*: 0.00761, *P* = 0.0185), rostral anterior cingulate (*Left*: 0.0123, *P* = 0.00887), rostral middle frontal (*Left*: 0.00869, *P* = 0.022), superior frontal (*Left*: 0.0109, *P* = 0.00218), superior parietal (*Left*: 0.00823, *P* = 0.0247; *Right*: 0.00931, *P* = 0.0162), superior temporal (*Left*: 0.00864, *P* = 0.00508; *Right*: 0.00607, *P* = 0.0485), supramarginal (*Left*: 0.00969, *P* = 0.00712; *Right*: 0.00772, *P* = 0.0311), transverse temporal (*Left*: 0.00695, *P* = 0.0431). When age × sex was included in the model, no ROI showed a significant AP × age effect. All *P*-values are FDR-corrected.

In initial sex-pooled analyses, the age × AP interaction was a significant predictor of cortical thickness in 40 predominantly frontoparietal ROIs, in line with prior reports (Fig. 1). Across these significant ROIs, higher AP scores were associated with reduced rates of age-related cortical thinning (β = 0.00594 to 0.0145, FDR-adjusted *P* < 0.05). Controlling for co-occurring internalizing or externalizing psychopathology yielded nearly identical findings (Fig. 1).

When an age × sex interaction term was included in the model, no ROI showed a significant age × AP effect. Across nearly all ROIs, addition of the age × sex term improved model fit relative to the original model (66/68 ROIs by Akaike Information Criterion; 63/68 ROIs by Bayesian Information Criterion). Notably, all 40 ROIs that were significant in the initial analysis showed improved model fit on both metrics when the age × sex interaction was included. In follow-up sex-stratified analyses, across all ROIs, the age × AP interaction was not significant in males (N = 5,782; 14,088 scans) or females (N = 5,311; 12,690 scans). Controlling for pubertal status did not meaningfully alter sex-stratified results. In a sex-pooled analysis, no ROI showed a significant three-way age × CBCL AP × sex effect.

**Fig. 2.**
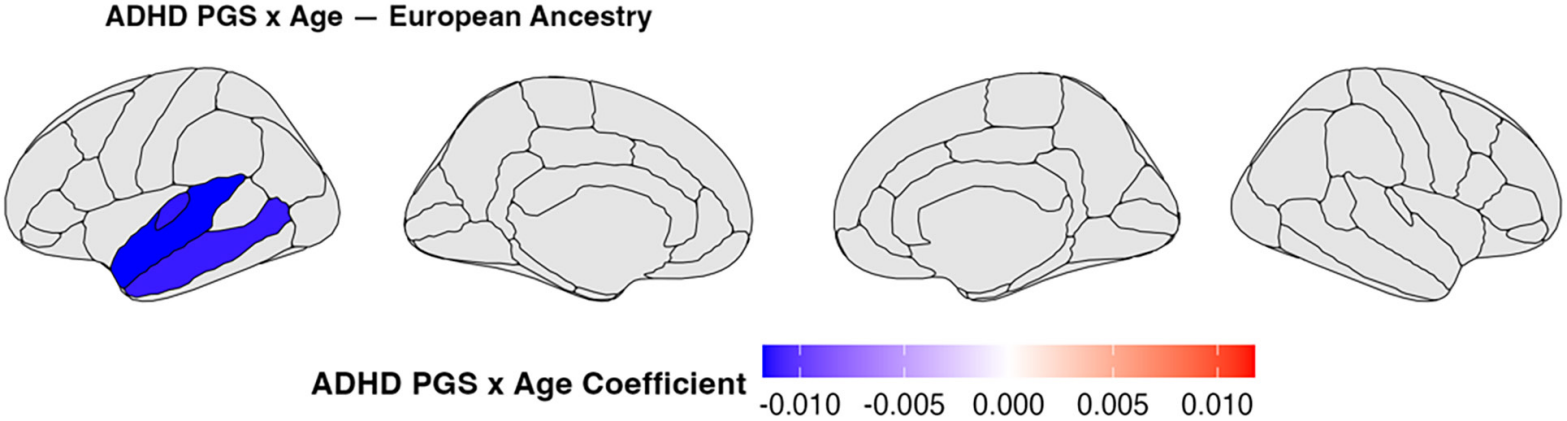
Shown above are the thresholded (corrected *P* < 0.05) coefficient maps for the age × PGS effect in the model run on the imputed European-like-ancestry only sample. three ROIs showed a significant age × PGS effect: middle temporal (*Left*: −0.0112, *P* = 0.0288), superior temporal (*Left*: −0.0118, *P* = 0.00985), transverse temporal (*Left*: −0.0113, *P* = 0.0473). All *P*-values are FDR-corrected.

In genetic analyses, the age × ADHD PGS interaction was a significant predictor of cortical thickness in 3 ROIs (Fig. 2, β = −0.0118 to −0.0112, FDR-adjusted *P* < 0.05). Contrary to the delayed maturation hypothesis, higher ADHD PGS was not associated with reduced rates of cortical thinning in any ROI. Instead, in all three significant ROIs, higher ADHD PGS was associated with accelerated age-related cortical thinning.

In a series of follow-up sensitivity analyses, we adopted a case–control design using ADHD diagnosis (derived from the parent version of the Kiddie Schedule for Affective Disorders and Schizophrenia–Computerized Version) in place of a dimensional, variable-centered approach, and reran all models with age specified as a quadratic term rather than a first-order linear effect. For the latter, some cortical regions showed modest improvements in model fit; however, deviations from first-order linear thinning were minimal. Additional details are provided in *SI Appendix*. Importantly, findings were highly consistent across all follow-up analyses: sex-stratified models revealed no evidence of an interaction between AP (or ADHD diagnosis) and age (modeled as either a linear or quadratic term) on cortical thickness.

## Discussion

For nearly two decades, delayed age-related cortical thinning has been widely viewed as a neurodevelopmental marker of attention-related psychopathology and ADHD. However, our findings suggest that this association may be largely confounded by sex differences in cortical development. Seminal studies on this topic relied solely on matching clinical and control groups with respect to sex composition, but did not model age × sex interactions (1), and subsequent work controlled for sex as a main effect but, similarly, did not include age × sex interaction terms (2, 3)—a critical limitation given more recently established sex differences in cortical thickness developmental trajectories (7).

Whereas our initial sex-pooled analysis revealed significant age × AP effects across predominantly frontoparietal regions—indicating reduced rates of age-related thinning at higher AP scores—these associations were dramatically attenuated when accounting for sex differences in cortical thickness trajectories. Across nearly all ROIs, inclusion of an age × sex interaction term resulted in better model fit. In follow-up sex-stratified analyses, the age × AP interaction failed to reach statistical significance in either males or females, further indicating that unaccounted for sex-related differences in neurodevelopment contributed to the age × AP effects in the sex-pooled model. Results from the age × ADHD PGS model were not consistent with ADHD symptomatology being associated with delayed cortical thinning. Instead, higher ADHD PGS was linked to accelerated cortical thinning across several ROIs. Critically, there was not a single ROI in which ADHD PGS was significantly associated with slowed age-related cortical thinning. These findings were robust across sensitivity analyses, including models using ADHD diagnosis and both linear and quadratic age terms, all of which yielded convergent results.

Taken together, these findings suggest that previously reported associations between attention problems and delayed cortical thinning are largely attributable to unaccounted-for sex differences in neurodevelopment. Our results call into question an influential framework in developmental neuroscience and psychiatry that has shaped clinical understanding of ADHD for nearly 20 y, underscoring that cortical maturation patterns should not be interpreted as biomarkers for attention-related psychopathology without rigorous accounting for sex-related variation in brain development.

## Materials and Methods

Experimental methods are provided in *SI Appendix*.

## Supplementary Material

Appendix 01 (PDF)

Code S01 (TXT)

## Data Availability

Tabular data have been deposited in ABCD Study (https://doi.org/10.82525/jy7n-g441) (8).
