# Supplementary material for: Attention problems and cortical maturation in a large longitudinal sample of youths: The importance of accounting for sex differences
Source: Proc Natl Acad Sci U S A. 2026 May 18;123(21):e2605729123. doi: 10.1073/pnas.2605729123 (PMC13213984; doi:10.1073/pnas.2605729123)
Supplement: Supplementary file 1 — Appendix 01 (PDF) [file pnas.2605729123.sapp.pdf]

PNAS

Supporting Information for:

Attention problems and cortical maturation in a large longitudinal sample of youths: The importance of accounting for sex differences

AUTHOR(S): Shannon D. O'Connor, MS[1]; Robert Loughnan, PhD[2,3]; Jonathan Ahern, BS[2,3]; Chun Chieh Fan, MD, PhD[2,4]; Robert R. Althoff, MD[1]; Hugh Garavan, PhD[1]; Alexandra Potter, PhD[1]; Matthew D. Albaugh, PhD[1]

Affiliations:

[1]Department of Psychiatry, University of Vermont, Burlington, Vermont, USA.
[2]Center for Population Neuroscience and Genetics, Laureate Institute for Brain Research, Tulsa, Oklahoma, USA.
[3]Department of Cognitive Science, University of California, San Diego, La Jolla, California, USA.
[4]Department of Radiology, University of California San Diego, La Jolla, California, USA.

Correspondence to Dr. Matthew D. Albaugh, Department of Psychiatry, Larner College of Medicine, University of Vermont, University Health Center campus, 1 South Prospect Street, Burlington, VT 05401

Email: malbaugh@uvm.edu

This file includes:

Supporting Methods

SI References

Supporting Methods

Sample description

The ABCD Study comprises 11,868 participants recruited at 9-10 years of age across 21 sites. Participants completed an MRI scan every 2 years (1). Additional details regarding the ABCD sample demographics and recruitment can be found in Garavan et al. (2). The present study utilized data from the ABCD Study 6.0 release, which includes data collected through January 2024, with MRI scan data from baseline, 2-year, 4-year, and 6-year assessments. After observations that were missing relevant covariates and excluding scans that failed quality control (QC), 27,172 scans from 10,730 unique participants were included in the primary analysis. The sample was 47.93% female and skewed towards higher income levels, with 42.15% of participants reporting annual household income greater than $100,000. In addition to the scan QC flags provided in the official data release, we imposed a surface hole number (SHN) threshold of 63, and included SHN as a covariate in all analyses, as recommended in Elyounssi et al (3). SHN for all scans were obtained through the ABCD consortium.

Our sample had a mean CBCL AP t-score of 55.54 (SD=5.75). At the baseline assessment, 134 (2.68%) females and 152 (2.84%) males had a CBCL AP t-score in the clinical range (>70), and 145 (2.90%) females and 308 (5.75%) males had a CBCL AP t-score in the borderline range (65-69). At the 2-year follow up assessment, 84 (2.47%) females and 81 (2.34%) males had a CBCL AP t-score in the clinical range, and 98 (2.89%) females and 149 (3.83%) males had a CBCL AP t-score in the borderline range. At the 4-year follow up assessment, 77 (2.89%) females and 49 (1.60%) males had a CBCL AP t-score in the clinical range, and 138 (5.18%) females and 104 (3.40%) males had a CBCL AP t-score in the borderline range. At the 6-year follow up assessment, 39 (2.16%) females and 21 (1.06%) males had a CBCL AP t-score in the clinical range, and 59 (3.29%) females and 63 (3.18%) males had a CBCL AP t-score in the borderline range.

There was a significant difference in AP scores by sex as well: males showed an average CBCL AP score of 3.17 (SD=3.59) and females showed an average CBCL AP score of 2.24 (SD=2.99) ($t=23.12$, $p<0.00001$). When fitting a linear model predicting CBCL AP raw score on age and sex, sex had a coefficient of -0.994 ($p<0.000001$, male as the reference) and age had a coefficient of -0.0836 ($p<0.000001$). This suggests that females generally have lower CBCL AP raw scores relative to males, and that CBCL AP raw score tends to decrease over age. When fitting a linear model predicting CBCL AP raw score on age, sex, and age x sex, age x sex had a coefficient of 0.102 ($p<0.00001$), suggesting that males show a faster decrease in CBCL AP raw score over age relative to females. Together, this suggests that males tend to have higher AP scores than females, and this effect is most pronounced at younger ages. Models were also constructed to see how puberty relates to CBCL AP in males and females, with CBCL as the outcome and parent-reported pubertal development, age, and household income as covariates, with participant nested in family for random effects. Parent-reported pubertal development did predict CBCL in females ($\beta=0.20$, $p=0.000014$), but not in males ($\beta=0.006$, $p=0.91$).

Among participants analyzed: 10,201 had data at baseline, 7,216 had data at the 2-year follow up, 5,668 had data at the 4-year follow up, and 3,764 had data at the 6-year follow up. There was an average of 2.4 time points per participant, with 2547 with 1 time point of available data, 3447 with 2 time points of available data, 3456 with 3 time points of available data, and 1760 with 4 time points of available data.

Longitudinal Stability of CBCL AP

In the sample, approximately 67% of total variance in AP was between-person (as indicated by the intra-class correlation coefficient from a linear mixed-effect model regressing AP on age), indicating that most of the variation in AP was attributable to stable between-subject differences. Between timepoint correlations were r=.71 (baseline to year 2), r=.68 (year 2 to year 4), and r=.72 (year 4 to year 6). Taken together, this level of stability is in line with attention problems frequently conceptualized as a trait-like characteristic and having a high degree of genetic heritability.

Primary Analyses

Associations between the Child Behavior Checklist (CBCL) Attention Problems (AP) scale raw score and region-level developmental changes in cortical thickness were estimated using the "lme4" R package and statistical inference was conducted using the "lmerTest" R package (4, 5). CBCL AP was included as a time-varying covariate in analyses. The initial model was specified as:

CT ~ Age + Sex + Household Income + AP + SHN + ICV + Age × AP + (1|Scanner ID/Family ID/Subject ID)

After fitting the model to all 68 regions-of-interest (ROIs) in the Desikan-Killiany atlas parcellation (6), p-values for the age × AP interaction were adjusted for multiple comparisons using a false discovery rate (FDR) correction.

Four additional models were constructed to assess if the age × AP effect was robust to each of the following: CBCL internalizing problems score, CBCL externalizing problems score, age x sex. The 3-way interaction age x CBCL AP x sex was also evaluated. Sex-stratified analyses, both with and without pubertal status, were also constructed, both with and without pubertal status included. Puberty was measured using the raw mean score from the parent-reported pubertal development scale (PDS) (7).

Genetic Analyses

Associations between ADHD polygenic risk score (PGS) and cortical development were similarly estimated using linear mixed-effects models. PGSs in the ABCD sample were determined using genetic information from saliva and blood (8) and assayed using the Smokescreen™ Genotyping array (9). Markers from the Smokescreen array were imputed using the TOPMED imputation server (10-12) to improve overlap with PGS posterior effects (13). Using a best guess threshold of 0.9, these imputed variants were converted to an integer number of alleles, and the target data were further restricted to only autosomal variants with a minor allele frequency of 1% using PLINK v2 (14). The ADHD PGS was derived from Demontis et al. (15) using pgs_calc (16) and is available via the PGS Catalogue, a curated open-source database (17). The ADHD polygenic risk score used in the present analysis has been previously shown to predict CBCL AP in the ABCD sample (18). The ADHD PGS model was specified as:

CT ~ Age + Sex + Household Income + ADHD PGS + SHN + ICV + Age × ADHD PGS + Ancestry Principal Components 1-16 + (1|Scanner ID/Family ID/Subject ID)

This model was run both in the full sample of participants with genetic data (N=10,310), and in only those of imputed European-like ancestry (N= 6,298) as estimated using SNP weights (19) and external genetic reference panels (20, 21). In response to a reviewer's concern, this latter analysis was restricted to participants of European-like ancestry to account for potential differences in ADHD PGS performance based on test-target sample ancestry alignment.

Additional Analyses

Age Nonlinearity

To assess the appropriateness of including age as a first-order linear effect, models were constructed modeling cortical thickness both as a function of age, sex, household income (6-level), ICV, and our nested random effects, and as a function of these same covariates along with an age-squared term. The age-squared term had a significant effect in 45/68 ROIs, and all 45 of these ROIs showed an improvement in Akaike information criterion (AIC) and a significant improvement based on likelihood ratio test with the inclusion of the age-squared term.

In all ROIs, we also assessed the impact of adding age x sex and age-squared x sex terms to the model that included age-squared and covariates only. The addition of these terms significantly improved model fit in 67/68 ROIs. In contrast, the addition of age x CBCL AP and age-squared x CBCL AP led to improved

model fit in 33/68 ROIs relative to the model with only age-squared and covariates and did not improve model fit in any ROI relative to the model with age x sex and age-squared x sex and covariates.

Despite only subtle differences from a linear trajectory even in ROIs where the addition of an age-square term improved model fit, we repeated primary analyses modeling age as a quadratic term. Specifically, in these 45 ROIs, the original sex-pooled model and the model with age x sex were rerun with the addition of an age-squared term (and age-squared interactions where applicable) along with an age-squared x CBCL AP interaction. In the initial age x CBCL AP and age-squared x CBCL AP model, 37 ROIs showed a significant age x CBCL AP effect, and none showed a significant age-squared x CBCP AP effect in the original model. Model fit was improved by AIC and likelihood ratio test in 39 ROIs (F-statistics ranging from 3.76 to 10.24 in ROIs with a significant improvement). In the model with age x sex included, no ROIs showed a significant age x CBCL AP effect, and only 1 ROI (left caudal middle frontal, F=6.76) showed a significant age squared x CBCL AP effect, though the inclusion of age x CBCL AP and age-square x CBCL AP did not significantly improve model fit in this ROI.

KSADS-COMP ADHD Diagnosis

Because seminal papers identifying delayed cortical thinning as a potential biomarker of ADHD relied on clinically diagnosed ADHD rather than a dimensional measure of AP, we additionally ran our primary analyses using a case-control design. In this approach, we used parent-reported KSADS-COMP current ADHD diagnosis as a proxy for a clinical diagnosis to define “cases” and “controls” in our sample. Our “controls” never had a current, past, full or partial remission KSADS-COMP ADHD diagnosis, nor did they ever have an unspecified or other attention disorder KSADS-COMP diagnosis. We defined “cases” in two ways: a “strict” method where cases had to have a current KSADS diagnosis at all available time points, and a “lenient” method where cases had to have a current KSADS diagnosis at all or all but one of their available time points. By the stricter definition, there were 365 cases (272/74.5% male, 93/25.5% female). For the more lenient definition, there were 755 cases (526/69.7% male, 229/30.3% female). There were 8887 (4329/48.7% male, 4558/51.3% female) controls. Cases were defined from non-SHN-QC filtered data, and scans with a SHN of 63 or higher were excluded after cases were defined.

We ran the following model:

CT ~ Age + Sex + Household Income + Case vs Control + ICV + Age $\times$ Case vs Control + (1|Scanner ID/Family ID/Subject ID)

We then ran an additional model that included age x sex. We ran our KSADS-based analyses in the full available sample of cases and controls, along with in a sex-balanced sample where female controls were under sampled to match the sex ratio of the cases group (resulting in N=5809 controls for the strict definition of cases and N=6214 controls for the lenient definition), and in a 1:1 matched on sex sample where both male and female controls were under sampled to match the exact number and sex balance of cases for both the strict and lenient definitions of cases.

Longitudinal coverage was also assessed for our KSADS-COMP cases vs controls. At baseline, there were 322 strictly defined cases, 688 leniently defined cases, and 8076 controls. At the 2-year follow up, there were 145 strictly defined cases, 437 leniently defined cases, and 5634 controls. At the 4-year follow up, there were 119 strictly defined cases, 302 leniently defined cases, and 4432 controls. At the 6-year follow up, there were 48 strictly defined cases, 168 leniently defined cases, and 2904 controls. Though there is significant participant drop-off at later time points, our number of even strictly defined cases at the 6-year follow up rivals that in Shaw et al (22), in which there were 223 cases and 223 controls at Time 1. 111 cases and 119 controls at Time 2, 59 cases and 59 controls at Time 3, and only 11 cases and 11 controls at Time 4.

Among controls, there was an average of 2.4 time points per participant, with 2085 participants with 1 available time point, 2779 participants with 2 available time points, 2689 with 3 available time points, and 1334 with 4 available time points. Among strictly defined cases, there was an average of 1.7 time points per participant, with 184 participants with 1 available time point, 113 participants with 2 available time points,

48 participants with 3 available time points, and 20 participants with 4 available time points. Among leniently defined cases, there was an average of 2.1 time points per participant, with 194 participants with 1 available time point, 336 participants with 2 available time points, 171 participants with 3 available time points, and 54 participants with 4 available time points.

There were no significant effects for age x case vs control for the stricter case definition in any sample or model variant. For the more lenient case definition, there were 15 ROIs with a significant age x case vs control effect in the original model in the full sample (βs ranging from 0.03036 to 0.05419, FDR-corrected $p<0.05$). There were no significant age x (lenient) case vs control effects in the model that included age x sex. There were also no significant age x (lenient) case vs control effects for any model in either the sex balanced or sex matched samples.

SI References

1. T. L. Jernigan, S. A. Brown, G. J. Dowling, The Adolescent Brain Cognitive Development Study. *J Res Adolesc* **28**, 154–156 (2018).
2. H. Garavan *et al.*, Recruiting the ABCD sample: Design considerations and procedures. *Developmental Cognitive Neuroscience* **32**, 16–22 (2018).
3. S. Elyounssi *et al.*, Addressing artifactual bias in large, automated MRI analyses of brain development. *Nature Neuroscience* **28**, 1787–1796 (2025).
4. D. Bates, M. Mächler, B. Bolker, S. Walker, Fitting Linear Mixed-Effects Models Using lme4. *Journal of Statistical Software* **67**, 1 – 48 (2015).
5. A. Kuznetsova, P. B. Brockhoff, R. H. B. Christensen, lmerTest Package: Tests in Linear Mixed Effects Models. *Journal of Statistical Software* **82**, 1 – 26 (2017).
6. R. S. Desikan *et al.*, An automated labeling system for subdividing the human cerebral cortex on MRI scans into gyral based regions of interest. *NeuroImage* **31**, 968–980 (2006).
7. A. C. Petersen, L. Crockett, M. Richards, A. Boxer, Pubertal development scale. *The Journal of Early Adolescence* (1988).
8. K. A. Uban *et al.*, Biospecimens and the ABCD study: Rationale, methods of collection, measurement and early data. *Developmental Cognitive Neuroscience* **32**, 97–106 (2018).
9. J. W. Baurley, C. K. Edlund, C. I. Pardamean, D. V. Conti, A. W. Bergen, Smokescreen: a targeted genotyping array for addiction research. *BMC Genomics* **17**, 145 (2016).
10. S. Das *et al.*, Next-generation genotype imputation service and methods. *Nature Genetics* **48**, 1284–1287 (2016).
11. D. Taliun *et al.*, Sequencing of 53,831 diverse genomes from the NHLBI TOPMed Program. *bioRxiv* 10.1101/563866, 563866 (2019).
12. C. Fuchsberger, G. R. Abecasis, D. A. Hinds, minimac2: faster genotype imputation. *Bioinformatics* **31**, 782–784 (2015).
13. D. Taliun *et al.*, Sequencing of 53,831 diverse genomes from the NHLBI TOPMed Program. *Nature* **590**, 290–299 (2021).
14. C. C. Chang *et al.*, Second-generation PLINK: rising to the challenge of larger and richer datasets. *GigaScience* **4** (2015).
15. D. Demontis *et al.*, Discovery of the first genome-wide significant risk loci for attention deficit/hyperactivity disorder. *Nature genetics* **51**, 63–75 (2019).
16. S. A. Lambert *et al.*, Enhancing the Polygenic Score Catalog with tools for score calculation and ancestry normalization. *Nature Genetics* **56**, 1989–1994 (2024).
17. S. A. Lambert *et al.*, The Polygenic Score Catalog as an open database for reproducibility and systematic evaluation. *Nature Genetics* **53**, 420–425 (2021).
18. B. B. Lahey *et al.*, Associations of polygenic risk for attention-deficit/hyperactivity disorder with general and specific dimensions of childhood psychological problems and facets of impulsivity. *Journal of psychiatric research* **152**, 187–193 (2022).
19. C.-Y. Chen *et al.*, Improved ancestry inference using weights from external reference panels. *Bioinformatics* **29**, 1399–1406 (2013).
20. A. Auton *et al.*, A global reference for human genetic variation. *Nature* **526**, 68–74 (2015).
21. D. Reich *et al.*, Reconstructing Native American population history. *Nature* **488**, 370–374 (2012).
22. P. Shaw *et al.*, Attention-deficit/hyperactivity disorder is characterized by a delay in cortical maturation. *Proceedings of the National Academy of Sciences* **104**, 19649–19654 (2007).
